# Focal Stroke in the Developing Rat Motor Cortex Induces Age- and Experience-Dependent Maladaptive Plasticity of Corticospinal System

**DOI:** 10.3389/fncir.2017.00047

**Published:** 2017-06-29

**Authors:** Mariangela Gennaro, Alessandro Mattiello, Raffaele Mazziotti, Camilla Antonelli, Lisa Gherardini, Andrea Guzzetta, Nicoletta Berardi, Giovanni Cioni, Tommaso Pizzorusso

**Affiliations:** ^1^Department of Neuroscience, Psychology, Drug Research and Child Health NEUROFARBA, University of FlorenceFlorence, Italy; ^2^Institute of Neuroscience, National Research Council (CNR)Pisa, Italy; ^3^Department of Clinical and Experimental Medicine, University of PisaPisa, Italy; ^4^Department of Developmental Neuroscience, IRCCS Stella Maris Scientific InstitutePisa, Italy; ^5^Institute of Clinical Physiology, National Research Council (CNR)Siena, Italy

**Keywords:** development, stroke, critical period, corticospinal system, maladaptive plasticity

## Abstract

Motor system development is characterized by an activity-dependent competition between ipsilateral and contralateral corticospinal tracts (CST). Clinical evidence suggests that age is crucial for developmental stroke outcome, with early lesions inducing a “maladaptive” strengthening of ipsilateral projections from the healthy hemisphere and worse motor impairment. Here, we investigated in developing rats the relation between lesion timing, motor outcome and CST remodeling pattern. We induced a focal ischemia into forelimb motor cortex (fM1) at two distinct pre-weaning ages: P14 and P21. We compared long-term motor outcome with changes in axonal sprouting of contralesional CST at red nucleus and spinal cord level using anterograde tracing. We found that P14 stroke caused a more severe long-term motor impairment than at P21, and induced a strong and aberrant contralesional CST sprouting onto denervated spinal cord and red nucleus. The mistargeted sprouting of CST, and the worse motor outcome of the P14 stroke rats were reversed by an early skilled motor training, underscoring the potential of early activity-dependent plasticity in modulating lesion outcome. Thus, changes in the mechanisms controlling CST plasticity occurring during the third postnatal week are associated with age-dependent regulation of the motor outcome after stroke.

## Introduction

Developmental stroke (perinatal or pediatric) represents a rare cerebrovascular disorder and a frequent cause of hemiplegia in children (Kirton, 2013; Kirton and deVeber, 2015). Despite the high plastic potential of developing brain, a unilateral ischemic lesion at early stage of development deeply affects the normal functional refinement of motor pathways (Eyre, 2007). Strengthening of corticospinal tracts (CST) ipsilateral projections from the healthy hemisphere (otherwise pruned during normal development), and weakening of the contralaterally projecting CST from the affected cortex, have been suggested to be the cornerstone of maladaptive plasticity mechanism after developmental injury (Eyre, 2007; Graziadio et al., 2012). The occurrence of this maladaptive plasticity could be responsible for driving the long term functional outcome towards a moderate or severe motor impairments (Martin and Lee, 1999; Eyre et al., 2001; Eyre, 2007; Cioni et al., 2011). Clinical findings have shown a correlation between structural and functional potentiation of ipsilateral gross white matter tracts and motor outcome in hemiplegic children (Eyre et al., 2001; Eyre, 2007; Cioni et al., 2011). However, a direct relationship between aberrant rewiring of gross white matter tract and axonal sprouting has not been determined, probably because of the limited resolution power achieved by currently available imaging techniques for human studies (Baek et al., 2013; Raffin and Dyrby, 2013). In this context, studies in animal models that allow detection of axonal sprouting may reveal to which degree injury affects plasticity of axonal terminal fields (Benowitz and Carmichael, 2010), and shed light on the molecular and anatomical basis of the maladaptive phenomenon. Axonal sprouting of survived neurons in adults upon lesion has been described as the principle adaptive plastic mechanism serving spontaneous and treatment-induced recovery (Carmichael, 2003; Murphy and Corbett, 2009; Jones et al., 2013; Wahl et al., 2014). However, recent findings in adult injured rats highlighted that interventions promoting a widespread and disorganized sprouting are detrimental (Wahl et al., 2014), suggesting that compensatory and adaptive axonal sprouting should follow precise location and branching “rules” to promote functional recovery.

During development, stroke induced sprouting can be also regulated by the timing of the lesion with respect to the stage of CST maturation. In rodents, CST reaches a maximum level of spinal cord gray matter innervations by P14 (Canty and Murphy, 2008). At this age, axon pruning gradually starts to select enduring connections according to a precise spatio-temporal expression of axon guidance molecules and synaptogenic factors (Weimann et al., 1999; Arlotta et al., 2005; Polleux et al., 2007). CST axon pruning continues over the following weeks, reaching the maximal levels at P21 (Hsu et al., 2006). Thus, we choose P14 and P21 to investigate whether a focal cortical stroke performed at these two ages, characterized by a different prevalence of CST sprouting and pruning, results in a different anatomical and behavioral response. Another important regulatory factor of CST maturation is suggested by studies showing that CST developmental refinement is strongly influenced by activity-dependent plasticity during a critical period conserved across different species (Martin et al., 2007, 2011; Friel et al., 2012). Therefore, we also studied whether skilled motor learning in lesioned animals can affect the lesion induced CST sprouting and motor outcome.

We found that behavioral outcome was dependent upon lesion timing: lesion in P14 rats induced more prominent impairments, both on general walking and skilled reaching abilities, than lesion at P21. Interestingly, poorer motor outcome in P14 lesioned animals was associated with an aberrant pattern of CST axonal sprouting from the healthy CST across gray matter laminae of the denervated spinal cord. Moreover, P14 lesion caused a contralesional reorganization of cortico-rubral pathway not observed when the lesion occurs 1 week later. Early skilled motor learning paradigm in P14 injured animals was sufficient to ameliorate fine and general motor outcome and to correct the age-dependent maladaptive sprouting pattern. These data suggest the existence of separate phases during the critical period of CST maturation wherein a cortical stroke can induce dramatically different outcomes at behavioral and anatomical level.

## Materials and Methods

### Anterograde Viral Tracing of CST

Cryoanesthetized P1–3 rat pups were injected with a total amount of 1 μl of AAV1-hSynap-eGFP-WPRE-bGH (titer 10^11^ copies/ml; Hutson et al., 2012) into four sites of right sensorimotor cortex (0.25 μl/site; coordinates from Bregma: 1.5 mm ML: 0.7 mm AP; 1 mm ML: 0.3 mm AP; 1 mm ML: 0.0 mm AP; 2 mm ML: 0.5 mm AP). Briefly, animals were fixed on a stereotaxic apparatus (Cunningham, Stoelting Co., Wood Dale, IL, USA) previously cooled with dry ice and viral injections were performed by using a pneumatic ejection pump (PDES-02TX, npi) attached with a pulled glass micropipette (30 μm tip). Pups were then placed under a heating lamp until they were fully awake and transferred to the home cage with their mothers.

### Induction of a Unilateral Focal Ischemic Lesion

P14 or P21 rats were administered intracortical injections of ET-1 (Sigma; 120 pmol/μL in sterile saline) as previously described (Gherardini et al., 2015). Briefly, animals were anesthetized by intraperitoneal injection of avertin (200 mg/Kg). Local injection of lidocaine (100 mg/Kg) under the scalp was provided. Body temperature was monitored and maintained at 37°C with a homothermic blanket (Harvard Apparatus Ltd, UK). Injections were performed at two sites of the left hemisphere corresponding to the primary motor area with respect to Bregma (1.5 mm ML: 0.0 mm AP; 1.5 mm ML: 0.5 mm AP; depth of 0.5 mm for P14 and 0.7 mm for P21 animals) by giving a total amount of 2 μl (240 pmol/μl) of ET-1 solution through a pulled glass micropipette connected to a manual pump at a rate of 0.5 μL/min with a 5-min interval before retracting the micropipette from the tissue. Sterile saline was injected in control groups with the same procedure. Immediately after surgery animals were given local antibiotic (aureomycin 3% cream) and analgesic (paracetamol, 10 mg/kg) administered in water. Animals were kept in a warming incubator until they recovered from anesthesia. P14 animals returned to their mothers in the home cage. All procedures were performed in accordance with the Italian Ministry of Health guidelines for care and maintenance of laboratory animals (law 116/92) and in strict compliance with the European Communities Council Directive 86/609/EEC. All the experiments were carried out in accordance with the directives of the European Community Council (2011/63/EU) and approved by the Italian Ministry of Health. Long Evans rats (*n* = 90) from P1 until P70–80 of both sexes were used.

### Anterograde Tracing of Cortico-Rubral Pathway

For the tracing of cortico-rubral connections, ET1 and control animals not previously transfected with AAV-GFP tracer were injected at P40 with the anterograde dye Biotinylated Dextran Amine (BDA). Rats were deeply anesthetized with avertin and placed in a stereotaxic frame. Animals were pressure-injected with 2 μl of a solution of 10% BDA (10,000 MW) in 0.01 M phosphate buffer, pH 7.2 into the forelimb mapping area of the contra-lesional hemisphere, 1 mm depth from pial surface at four sites (0.5 μl each point; stereotaxic coordinates: A-P 0, M-L 3 mm; A-P 0; M-L 2.5 mm; A-P 2 mm, M-L 2.5 mm; A-P 2.5 mm, M-L 2.5 mm).

### Functional Assessment

The effect of lesion-timing on long-term motor outcome was evaluated by general and fine motor behavioral tests performed in adult age, starting from P59 (see Supplementary Methods). An operator blind to assigned experimental group carried out all behavioral tests. Gait analysis to evaluate locomotion was performed by using a modified protocol from de Medinaceli et al. (1982), Metz and Schwab (2004) and Patterson et al. (2010). Sensorimotor coordination was assessed at vertical ladder according to Metz and Whishaw (2009), whereas muscle strength was measured by grip test (Doeppner et al., 2014). Reaching skills were daily tested from P60 by Montoya Staircase Test (MST) for 1 week (Montoya et al., 1991), skilled motor impairments were scored according to Smith et al. (2007) and Soleman et al. (2012) and expressed as percentage values.

### Early Skilled Learning Protocol

To investigate the effects of an early activity-dependent modulation of CST plasticity after developmental lesion, a group of animals treated with ET-1 at P14 were trained twice/day in the MST apparatus till they reached the plateau level of skilled motor learning, starting from P21. The effect of this training on long-term motor outcome was assessed at P59 as described before and results were compared with those of Saline or ET1 P14 injected animals.

### Ischemic Lesion Characterization

After 3 or 7 days after lesion induction, after behavioral assessment, or after 3 weeks following BDA injections (P60), animals were lethally anesthetized and transcardially perfused with 4% (PFA) 0.1 M phosphate buffer. Both brain and spinal cord were extracted and post-fixed overnight at 4°C, then immersed in a dehydrating solution of sucrose 30% and sodium azide 0.05% for at least 3 days. Dehydrated tissues were embedded with Tissue-Tek® OCT™, snap-frozen in −80°C cooled isopentane, and 50 μm coronal sections of brain and cervical spinal cord were cut with a cryostat (Leica).

### Cortical Lesion Measurements

Coronal sections of entire motor cortex were collected one out of two slices and then stained with propidium iodide (5 μg/μl) Sigma-Aldrich® and mounted and cover slipped. Mosaic images (2557 × 1917 pixels) were acquired with a fluorescence microscope (Axio Imager.Z2, Zeiss) using 5× EC-PLAN-NEOFLUAR objective (N.A. 0.25). Lesion area was measured using Image-J software. The volume was computed by multiplying individual lesion areas by the interslice distance.

### Analysis of Axonal Sprouting at Red Nucleus Level

For brains derived from animals injected with BDA, one every three slice were collected in the area of the tracer injection site, whereas all consecutive sections including the red nucleus were collected and then serially mounted on gelatine-coated slides. Slides were washed three times for 15 min in TBS-TX 0.3% pH 8.0 before incubation overnight with an avidin–biotin–peroxidase complex diluted in TBS-TX 0.3% (ABC elite; Vector Labs, Burlingame, CA, USA). The following day the slides were washed three times and rinsed with TBS-TX 0.3% for 30 min, and then for 5 min with 0.05 M Tris-HCl pH 8.0. Finally the tissue was stained using nickel-enhanced diaminobenzidine (DAB) protocol on slides (Vector Labs, Burlingame, CA, USA). Images were acquired with transmitted light microscope (Leica) and the area of interest (ROI), identified using the stereotaxic coordinates of the atlas of Paxinos and Watson (1998), was analyzed with the software Stereo Investigator (MBF Bioscience). A grid (2.5 mm^2^ each square) was superimposed on each of section and cortico-rubral projections to the ipsilateral and contralateral red nucleus were analyzed quantitatively in three squares (7.5 mm^2^) on all sections. Two parameters were taken into account: the “innervation index” and the “midline crossing fibers index” (Z’Graggen et al., 2000). Innervation index was calculated as ratio of fiber density of the ipsilesional (denervated) and contralesional (innervated) red nucleus. Midline crossing fibers index was quantified by counting BDA-positive fibers crossing the midline on each section and corrected for the inter animal tracing variability by dividing the number of midline crossing fibers per the total CTS axons fibers quantified into a ROI of cerebral peduncle (7.5 mm^2^).

### Viral Labeling Quantification in Spinal Cord

One every 10 slices of medullar pyramids (at the level of caudal cerebellum) were collected for quantification of the viral tracer labeled axons amount. Cervical spinal cord was sectioned from C6 to C8 levels for analysis of CST sprouting; a total number of four sections/level were obtained, collecting one every six-eight consecutive slices.

### Quantification of Green Fluorescent Protein Positive (GFP^+^) Bulk of Dorsal (dCST) and Ventral (vCST) Funiculi

To quantify the bulk of the labeled dorsal CST (dCST) and ventral CST (vCST), 10× magnification (EC-PLAN-NEOFLUAR objective, N.A. 0.3) images were acquired at fluorescence microscope (Axio Imager.Z2, Zeiss) and integrated fluorescence density of the labeled CST was computed by ImageJ software. For each animal, at least three sections per level were analyzed. Appropriate exposure parameters were chosen in order to avoid saturation of the highly intense signals coming from the funiculi and to minimize such variability in signal detection. To correct for inter animal tracing variability integrated density of both dCST and vCST were corrected by the number of GFP^+^ fibers counted in at least three section of the medullar pyramids (see Supplementary Material).

### Analysis of Axonal Sprouting at Spinal Cord Level

GFP^+^ axonal sprouting from labeled dCST and vCST was analyzed by a fluorescence microscope (Leica), magnification 20×, using the software Stereo Investigator (MBF Bioscience). At least three sections per level were measured in each rat. For all slices, GFP signal was detected without immunohistochemistry procedures. GFP^+^ axonal fibers originating from labeled dCST and reaching contralateral denervated spinal cord were counted as fibers crossing the spinal cord midline (M) and extending through four different distance lines (D1–4), with each line being 150 μm apart and spanning across all gray matter. GFP^+^ axonal fibers arising from labeled vCST and reaching ipsilateral denervated spinal cord were counted as fibers crossing the boundary between white-gray matter of the medial part of ventral horn (Wahl et al., 2014). Axonal sprouting was normalized on the total number of GFP labeled corticospinal axons counted on at least three coronal sections of medullar pyramids (see Supplementary Material).

### Laminar Pattern Distribution of Sprouted Axons

Z-stack images of three C6–C8 spinal cord sections per animal were scanned using fluorescence microscope (Axio Imager.Z2, Zeiss) and by applying structured illumination provided by Apotome.2 (Zeiss). 10X mosaic images (EC-PLAN-NEOFLUAR objective, NA 0.3) were acquired both in bright field and in green channel fluorescence, in order to recognize anatomical landmarks of spinal cord gray matter. Maximum intensity projections (MIPs) were generated from the group of ≅10 consecutive stacks yielding the higher mean pixel intensity. Semi-automated analysis for GFP^+^ axons detection and characterization was performed using a custom-made MATLAB® algorithm. Briefly, to sample all spinal cord laminae, 15 square ROIs (129 × 129 μm) per section covering all the dorso-ventral axes of spinal gray matter were selected using bright field cues (Supplementary Figure S2). GFP^+^ axons signal was therefore detected contemporary in the green channel. ROIs sampling was carried out in a standard fashion for all analyzed images. ROIs 1–5 identify dorsal sensory laminae (1–6), ROIs 6–10 the intermediate lamina 7 and ROIs 11–15 the ventral, motor laminae 8 and 9. Once selected, each ROI was processed for axons detection and measurement of their laminar pattern distribution. This parameter named “axonal complexity index” (ACI) refers to:

Axonal Complexity Index = Na * Ca

where Na is the mean number of detected axons per laminae (normalized for the number of GFP^+^ axons in medullar pyramids) and Ca is the mean axonal complexity per laminae (sum of branch points and endpoints per object). For further information about the algorithm see Supplementary Material.

### Statistical Analysis

All data are presented as mean ± standard error of the mean (SEM). *n* denotes number of sampling in each group. Significant level was settled as 0.05. Lesion volume measures, general motor behavior, analysis of sprouting at red nucleus level, fluorescence integrated density of CST, sprouting from vCST and laminar distribution were analyzed using *t*-test, Two-Way ANOVA or One-Way ANOVA, when appropriate. Normality assumption was validated using Shapiro-Wilk test; when rejected, data were transformed on ranks and then analyzed with the appropriate statistic test. Number of GFP^+^ axons on medullar pyramids was analyzed through non-parametric Kruskal-Wallis test. Behavioral data of MST and axonal sprouting from dCST were analyzed using Two-Way repeated measure ANOVA, with respectively number of days and sequential distances as within subject factors. *Post hoc* multiple comparison using Holm-Sidak test was performed, when appropriate. SigmaPlot®12.0 was used to perform the analysis. Size of experimental groups is shown in Table 1.

**Table 1:** Size of experimental groups.

Test∖Group	SAL-P14	ET1-P14	SAL-P21	ET1-P21	ET1-P14 TRAINING
Lesion volume	6	7	5	6	5
Lesion volume 3 days	–	3	–	5	–
Lesion volume 7 days	–	3	–	5	–
Vertical ladder test	10	11	8	9	5
Grip test	11	15	7	9	5
Gait test	11	9	7	9	5
Staircase test	15	13	10	16	5
Anatomical analysis (Spina cord)	5	6	4	6	5
Anatomical analysis (Red nucleus)	4	4	3	3	–

## Results

### ET-1 Injection in Forelimb Motor Cortex (fM1) Induces a Similar Damage in P14 and P21 Rats

**Figure 1:**
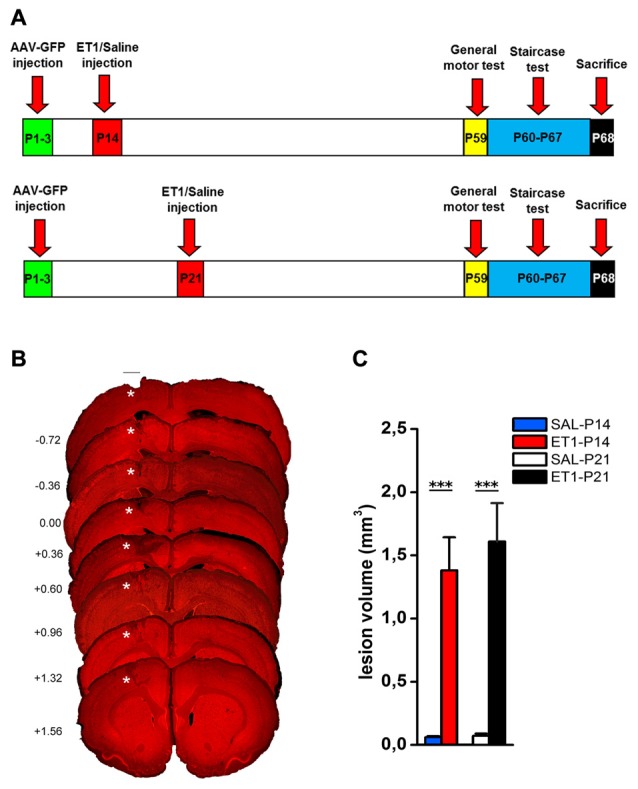
ET1 ischemic injury provoked in developing rats. **(A)** Experimental design. Rat pups were injected with AAV-green fluorescent protein (GFP) anterograde tracer, then lesioned at P14 or P21. Control animals were injected with saline. Once adults, animals performed behavioral tests and finally sacrificed. **(B)** Images sampled along the A-P axis (A-P level on the left hand side) showing ET1 ischemic lesion of the forelimb area. Lesion was induced at P21 and the rat was perfused at P70. Asterisks denote ET1 lesion. Scale bar, 1 mm. **(C)** Lesion volume quantified in ET1-P14/SAL-P14 and ET1-P21/SAL-P21 groups. Data are expressed as mean ± standard error of the mean (SEM). Asterisks denote significant differences respectively between ET1-P14 vs. SAL-P14 and ET1-P21 vs. SAL-P21. ***p < 0.001.

To induce a focal ischemic stroke, we performed intracortical injections of ET-1 (Gherardini et al., 2015) targeting the primary forelimb motor area (fM1) corresponding to the caudal forelimb area (CFA). We first assessed whether the stroke induction protocol adopted (Figure 1A) caused the same tissue damage when performed at P14 or P21. Nuclear staining with propidium iodide showed the presence of consistent tissue loss extending from the pia to the white matter (Figure 1B) in lesioned animals once adults. Quantitation in Figure 1C revealed that no difference in lesion volume was present between ET1-P14 (*n* = 7) and ET1-P21 (*n* = 6) rats (Two-Way ANOVA, factor age *p* = 0.588). As expected, lesion was almost absent in control rats injected with saline (Figure 1C, factor lesion *p* < 0.001). The lesion size was not different between ET1-P14 and ET1-P21 rats also at shorter times after lesion (3 and 7 days after lesion, Supplementary Figure S2). Thus, our protocol of stroke induction caused similar tissue damage at P14 and at P21.

### Timing of ET-1 Lesion during Development Determines Long-Term Motor Outcome

**Figure 2:**
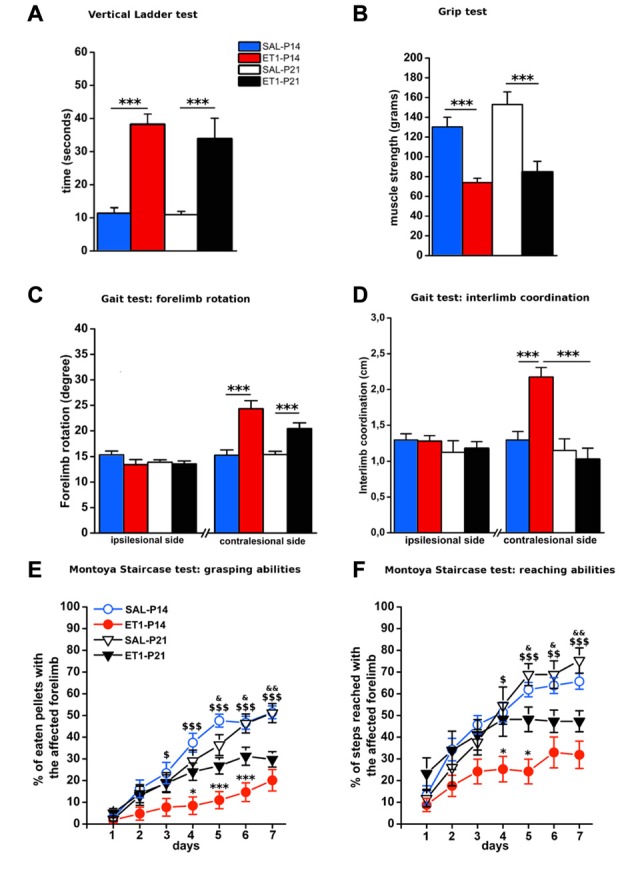
Timing of ET1 lesion during development determines long-term motor outcome. Age-independent effect of ET1 lesion on sensorimotor coordination **(A)**, muscle strength **(B)** and abduction of affected forelimb **(C)**. Age-dependent effect of ET1 lesion on interlimb coordination **(D)**, success rates in the grasping task **(E)** and reaching abilities **(F). (A–D)** Asterisks denote significant differences between groups, ***p < 0.001. **(E,F)** Asterisks denote significant differences between ET1-P14 and ET1-P21, *p < 0.05; ***p < 0.001. $ denotes significant differences between ET1-P14 and SAL-P14, ^$^*p* < 0.05; ^$$^*p* < 0.01; ^$$$^*p* < 0.001. & denotes significant differences between ET1-P21 and SAL-P21, ^&^*p* < 0.05; ^&&^*p* < 0.01. **(A–F)** Data are expressed as mean ± SEM.

We then evaluated the effect of lesion timing on motor outcome. Both general and skilled motor behavior was measured when animals reached young-adult age (P59; Figure 1A). Both at P14 and at P21, ET1 lesion affected motor function and muscle strength, indeed ET-1 animals performed worse than control animals in ladder climbing (Figure 2A, factor age *p* = 0.924, factor lesion *p* < 0.001) and grip test (Figure 2B, factor age *p* = 0.070, factor lesion *p* < 0.001).

Intriguingly, analysis of locomotor behavior and skilled reaching ability revealed more pronounced deficits for rats lesioned at P14. Indeed, ET1-P14 animals had worse fore-hindlimb coordination with their affected contralesional side with respect to SAL-P14 and ET1-P21 animals (Figure 2D, right, factor lesion × age *p* = 0.002; ET1-P14 vs. SAL-P14, *p* < 0.001, ET1-P14 vs. ET1-P21 *p* < 0.001). By contrast forelimb rotation analysis revealed a significant abduction of the lesioned (contralesional) forelimb vs. controls (Figure 2C, right, factor lesion *p* < 0.001), but without any effect of lesion timing (Figure 2C, right, factor age *p* = 0.114). No alteration was observed on the ipsilesional forelimb both for limb rotation (Figure 2C, left, interaction lesion × age *p* = 0.304) and for interlimb coordination (Figure 2D, left, interaction lesion × age *p* = 0.735).

Assessing skilled reaching ability using the MST revealed a deleterious effect of lesion age. Grasping deficits were more prominent in ET1-P14 animals than in ET1-P21 (Figure 2E, factor lesion × age *p* = 0.02, *p*-values in figure legend). Moreover, motor task learning was also affected in an age-dependent manner. In fact, whilst ET1-P21 and control groups begin to show significant task learning starting respectively from 2nd to 3rd day of testing (Figure 2E, factor day *p* < 0.001, 2nd vs. 1st day within SAL-P14 *p* = 0.001; 3rd vs. 1st day within SAL-P21 *p* < 0.001; 3rd vs. 1st day within ET1-P21 *p* = 0.001), ET1-P14 animals showed a delayed learning of the task, with significant learning only present from day 6th (factor day *p* < 0.001, 6th vs. 1st day within ET1-P14 *p* = 0.02).

A comparable trend was observed when reaching and extension abilities of the affected forelimb were quantified by counting the number of steps reached for pellet retrieval. Although lesion induced an overall worsening with respect to controls (Figure 2F, factor group × age *p* < 0.001), P14 lesioned animals had a stronger long-term reaching impairment than P21 injured rats (factor lesion × age *p* = 0.041, ET1-P21 vs. ET1-P14 *p* < 0.05). Furthermore, learning curve comparisons confirmed the worse ET1-P14 extension ability. Indeed, animals started to learn the reaching motor task from the 6th day of the test whereas the other groups were able to learn from 2nd to 3rd day of test (factor day *p* < 0.001, 6th vs. 1st day within ET1-P14, *p* < 0.001; 2nd vs. 1st day within SAL-P14 *p* < 0.001; 3rd vs. 1st day within SAL-P21 *p* < 0.001; 3rd vs. 1st day within ET-1 P21 *p* = 0.004). No statistical difference was observed between SAL-P14 and SAL-P21 controls either in grasping (SAL-P14 vs. SAL-P21, *p* = 0.81) or in reaching performance (SAL-P14 vs. SAL-P21, *p* = 0.62).

### ET-1 Lesion in Developing Rats Causes an Age Dependent Maladaptive Rewiring of Cortico-Rubral and Cortico-Spinal Tracts

**Figure 3:**
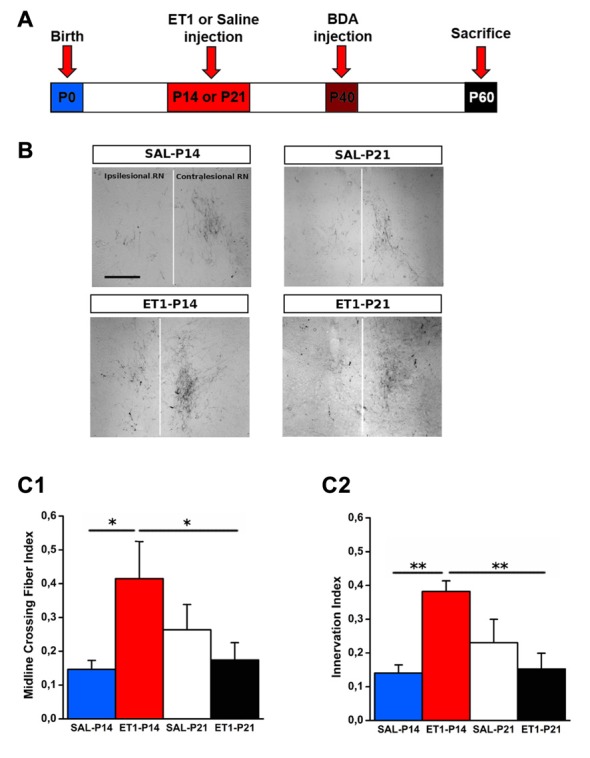
Age-dependent effect of lesion on cortico-rubral plasticity. **(A)** Experimental design. Rats were lesioned at P14 or P21, then were injected at P40 in the contralesional cortex with biotinylated dextran amine (BDA) anterograde tracer, and finally sacrificed at P60. **(B)** Representative 10× magnification images of innervated (contralesional) and denervated (ipsilesional) red nucleus from animals of different groups. Vertical white line indicates midline. Scale bar, 200 μm. **(C1)** Quantification of BDA-labeled axons projecting to the denervated side of red nucleus across the midline (average from at least four sections per case). **(C2)** Ratio of fiber density of the denervated (ipsilesional) and innervated (contralesional) red nucleus (averaged from at least four sections per case). **(C1,C2)** *p < 0.05; **p < 0.01. Data are expressed as mean ± SEM.

To compare the structural consequences of P14 and P21 focal cortical stroke, we first analyzed sprouting of cortico-rubral fibers originating from the contralesional hemisphere in the red nucleus that is involved in the fine control of reaching movement (Z’Graggen et al., 2000; Williams et al., 2014). Axons were labeled with BDA at P40 and analyzed at P60 in P14 and P21 ET1 lesioned and control unlesioned rats (Figure 3A). We first counted fibers crossing the midline: ET1-P14 lesioned rats showed a significant increase in the number of BDA-labeled axons crossing the midline with respect to SAL-P14 (Figures 3B,C1, factor treatment × age *p* < 0.05, ET1-P14 vs. SAL-P14, *p* = 0.024) and ET1-P21 lesioned animals (ET1-P14 vs. ET1-P21, *p* < 0.05). We also computed an innervation index that represents the labeling density ratio between the ipsilesional (denervated) and the contralesional (innervated) nucleus. Also for this index we observed a significant increase in ET1-P14 lesioned animals vs. SAL-P14 and ET1-P21 group (Figures 3B,C2, factor treatment × age *p* = 0.004; ET1-P14 vs. SAL-P14 *p* = 0.003, ET1-P14 vs. ET1-P21 *p* = 0.001). These data strongly suggest that an early focal ischemia induce a contralesional reorganization of cortico-rubral pathway, that is not observed when the lesion occurs 1 week later.

We then decided to analyze CST sprouting at cervical spinal cord level. In this analysis, we evaluated not only the amount of sprouting, but also the precise localization of the newly formed fibers, a crucial aspect for correlation with behavioral outcome (Wahl et al., 2014). Thus, we decided to use a viral labeling technique (Okada et al., 1999; Spergel et al., 2001; Harvey et al., 2009; Alves et al., 2014) that had more temporal stability and did not require another post-lesion surgery session.

**Figure 4:**
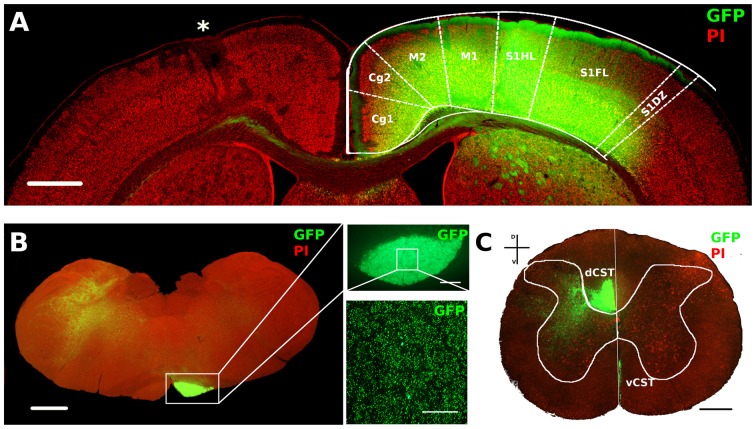
AAV1-GFP viral labeling of contralesional corticospinal tracts (CST). **(A)** Representative image of motor cortex from a P14 lesioned animal characterized by lesion (*) on one hemisphere and a strong GFP signal in deep layers of the opposite cortex. Cortical areas were outlined according to Paxinos and Watson atlas. Scale bar, 1 mm. **(B)** Mosaic image of caudal medulla oblongata containing GFP labeled medullar pyramid. Scale bar, 1 mm. Insets: 5× (upper) and 63× (bottom) magnifications of GFP^+^ axons in medullar pyramid. Scale bars, 100 μm and 50 μm, respectively. **(C)** Coronal section of C6 spinal cord stained with PI. Vertical white line indicates dorso-ventral midline. Note the ventral part of dorsal funiculus (dorsal CST, dCST) and the medial part of ventral funiculus (ventral CST, vCST) labeled with GFP. Scale bar, 500 μm.

**Figure 5:**
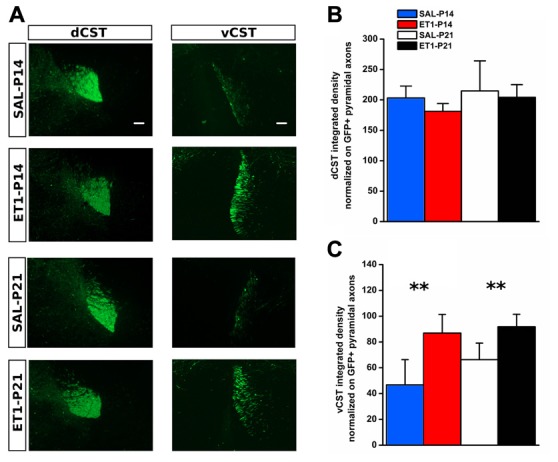
Developmental ET1 lesion prevents pruning of ipsilateral vCST in an age-independent manner. **(A)** Representative 10× magnification images of dCST (left) and vCST (right) from animals of different groups. Scale bar, 100 μm. Analysis of GFP^+^ signal from dCST **(B)** and vCST **(C)** funiculi was performed on non-saturated images: brightness and contrast parameters were optimized for detection of dCST and vCST bulks respectively, preventing visualization of axonal sprouts into the gray matter of denervated spinal cord. **(B)** No differences between groups. **(C)** Both ET1 lesioned groups had a greater ipsilateral ventral funiculus vs. control animals, suggesting that, at both ages, lesion affects developmental pruning. **p < 0.01. **(B,C)** Data are expressed as mean ± SEM.

We used AAV1-GFP viral vector injected into the fM1 to anterograde trace axons originating from pyramidal neurons (Figure 4A and Supplementary Figure S3) of the uninjured cortex. GFP^+^ axons were easily detected at the level of pyramidal medulla, although we also distinguished the presence of spared cortico-reticular and cortico-vestibular projections (Figure 4B). Moreover, dCST on the opposite side, and vCST on the same side of AAV-GFP cortical injections, were clearly observed in coronal sections of spinal cord (Figure 4C). We quantified fluorescence (GFP^+^) integrated density of dCST and vCST funiculi at C6–C8 spinal cord level to verify whether timing of developmental lesion might differently interfere with normal maturation of contralesional CST (Figure 5A). No significant difference in dCST fluorescence integrated density was present between all groups (Figure 5B, factor treatment × age *p* = 0.385), confirming that labeling occurred similarly in all AAV injected animals. On the contrary, vCST fluorescence integrated density of ET1-P14 and ET1-P21 was significantly higher with respect to control (SAL-P14 and SAL-P21) rats (Figure 5C, factor treatment *p* = 0.003), but no differences were detected between rats lesioned at different ages. These data indicate that pruning of vCST that normally occurs during development is prevented by lesioning the contralateral cortex, and are in agreement with the presence of a competition for connecting with spinal cord targets between axons coming from ipsilateral and contralateral hemispheres. These results parallel clinical evidence obtained in hemiplegic cerebral palsy (CP) patients showing that axonal diameter of ipsilateral, but not contralateral, corticospinal axons was increased with respect to healthy controls (Eyre, 2007).

**Figure 6:**
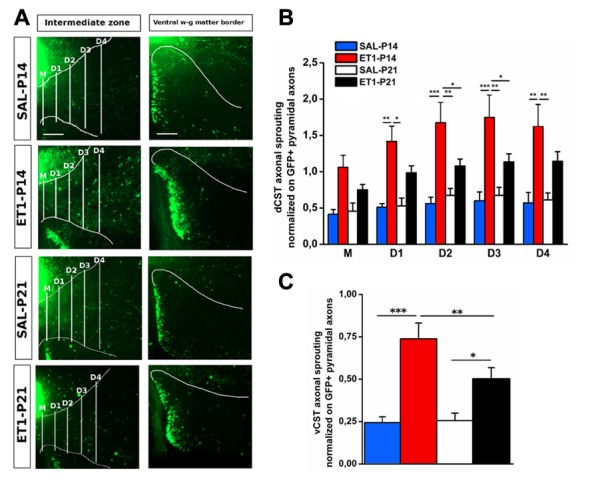
Lesion timing determines patterns of axonal sprouting in an age-dependent manner. **(A)** Representative images of denervated side of spinal cord showing dCST and vCST axonal sprouting towards spinal gray matter. Scale bars 200 μm. **(B)** dCST axonal sprouting. ET1-P14 animals show a greater degree of axonal sprouting from GFP^+^ dCST compared to ET1-P21 rats. Two-Way repeated measure ANOVA, factor group × distance *p* < 0.001, *post hoc* Holm-Sidak test. Asterisks indicates significance between groups: *p < 0.05, **p < 0.01, ***p < 0.001. **(C)** vCST axonal sprouting. ET1-P14 animals showed a greater number of axons from GFP^+^ vCST crossing white-gray matter border vs. ET1-P21 rats. Two-Way ANOVA, factor treatment × age *p* < 0.001, *post hoc* Holm-Sidak. Asterisks indicates significance between groups: *p < 0.05, **p < 0.01, ***p < 0.001. **(B,C)** Data are expressed as mean ± SEM.

We further assessed sprouting from dCST and vCST into the denervated C6–C8 spinal cord gray matter. GFP^+^ axons sprouting from uninjured dCST were counted from midline (M) to different distances (up to 600 μm medio-lateral in four steps 150 μm apart) towards denervated cervical gray matter (Figure 6A, left panels). Interestingly, ET1-P14 rats had a significant greater number of GFP^+^ sprouted axons than SAL-P14 and ET1-P21 groups, with elongation significantly extending beyond 300–450 μm from midline (Figure 6B, factor group × distance *p* = 0.006, *post hoc* *p* < 0.05). The number of GFP^+^ fibers originating from vCST and crossing the border between white and gray matter in the ventral horn of spinal cord was also counted (Figure 6A, right panels). Similarly to dCST, ischemic lesion induced spontaneous structural plasticity also from vCST (Figure 6C, factor treatment *p* < 0.001). Intriguingly, in ET1-P14 rats vCST sprouting was significantly more prominent than in ET1-P21 animals (Figure 6C, factor treatment × age *p* < 0.05, *post hoc* *p* = 0.006).

Since ET1-P14 lesioned rats displayed worse motor outcome but widespread axonal sprouting, we analyzed whether lesion induced sprouting targeted the correct laminae in the denervated spinal cord. The distribution of axonal sprouts on different spinal cord laminae was measured using a semi-automated algorithm (Supplementary Figure S1, see “Material and Methods” Section and Supplementary Material) computing, for each position in the spinal cord, an index designed “ACI” combining the number of axons detected and their branching measured by summing the number of branch points and endpoints.

**Figure 7:**
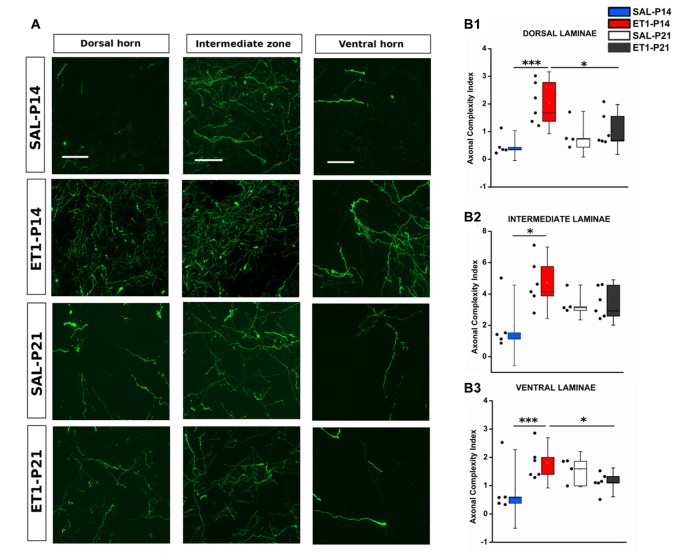
Mistargeted sprouting of CST after P14 lesion. **(A)** Micrographs showing different sprouting patterns of GFP^+^ fibers from the contralesional cortex towards dorsal (left), intermediate (middle) and ventral (right) zones of the denervated cervical spinal cord (C7) in different groups. Scale bars, 50 μm. **(B1–B3)** ET1-P14 animals exhibits a higher axonal complexity index (ACI) in dorsal and ventral laminae compared to controls and ET1-P21 animals. **(B1)** Two-Way ANOVA on ranks for dorsal laminae, factor treatment × age *p* < 0.01, *post hoc* Holm-Sidak. **(B2)** Two-Way ANOVA on ranks for intermediate lamina, factor treatment × age *p* < 0.05, *post hoc* Holm-Sidak. **(B3)** Two-Way ANOVA on ranks for ventral laminae, factor treatment × age *p* < 0.01, *post hoc* Holm-Sidak. Asterisks indicates significance between groups: *p < 0.05, ***p < 0.001. **(B1–B3)** Data are expressed as mean ± SEM.

In normal rats the intermediate laminae received the great majority of CST axons. However, ET1-P14 rats showed enhanced sprouting with respect to P21 ET1 rats only in dorsal and ventral laminae but not in the intermediate laminae (Figures 7A–B3). Indeed, ET1-P14 rats exhibited a significant increase in ACI compared to ET1-P21 animals in dorsal (Figure 7A left panels and B1, factor treatment × age *p* = 0.006, *post hoc* between ET1 groups *p* < 0.05), and in ventral laminae (Figure 7A right panel and B3, factor treatment × age *p* = 0.009, *post hoc* between ET1 groups *p* < 0.05). Interestingly, there was no significant difference of ACI in intermediate laminae between ET1-P14 and ET1-P21 animals (Figure 7A middle panels and B2, factor treatment × age *p* = 0.049, *post hoc* between ET1 groups *p* = 0.146) indicating that the lamina normally targeted by CST axons does not receive more sprouts in P14 lesioned than in P21 lesioned rats. Furthermore, whilst ACI did not change across laminae between ET1-P21 and SAL-P21 animals (Figures 7B1–B3, ET1-P21 vs. SAL-P21 *p* > 0.05 for all laminae), there was a significant increase of ACI between ET1-P14 and SAL-P14 in all laminae (Figures 7B1–B3, ET1-P14 vs. SAL-P14 *p* ≤ 0.01 for all laminae). These observations support the idea that cortical stroke at P14 causes a widespread sprouting of CST invading territories that are not normally involved in CST control of spinal circuits.

### Early Motor Training Restores Normal CST Refinement, Sprouting Pattern and Laminar Distribution in ET-1 P14 Animals

**Figure 8:**
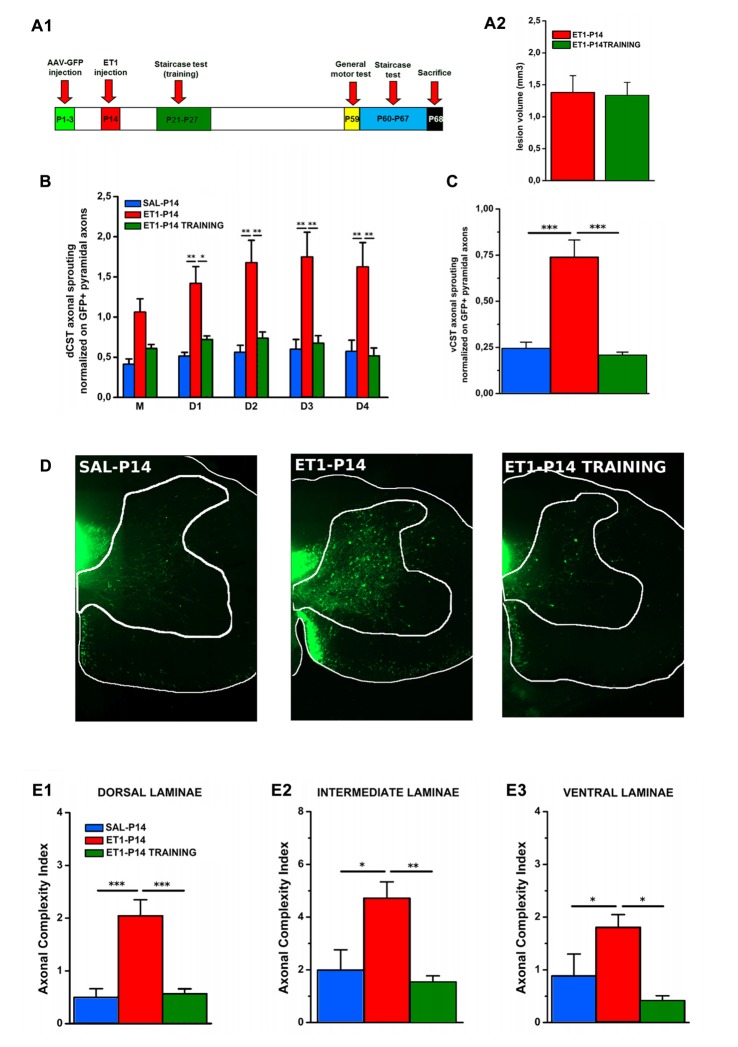
Early skilled motor learning corrects maladaptive CST rewiring after ET1-P14 lesion. **(A1)** Experimental design. **(A2)** Lesion volumes quantified in ET1-P14/ET1-P14 TRAINING groups, *t*-test, *p* > 0.05. **(B)** dCST axonal sprouting. ET1-P14 TRAINING group showed a significant reduction of aberrant spontaneous sprouting to the same levels as controls. Two-Way repeated measure ANOVA, factor group × distance *p* < 0.001, *post hoc* Holm-Sidak test. **(C)** vCST axonal sprouting. ET1-P14 TRAINING group showed a significant reduction of number of axons from GFP^+^ vCST crossing white-gray matter border vs. ET1-P14 rats and the same level of innervation as controls. One-Way ANOVA *p* < 0.001, *post hoc* Holm-Sidak. **(D)** Mosaic images representing the effect of early training on different sprouting patterns of GFP^+^ fibers from the contralesional cortex towards the denervated cervical spinal cord (C6) of different groups. Scale bars, 200 μm. **(E1–E3)** Early skilled motor learning promoted a significant drop in ACI of sprouted axons in ET1-P14 TRAINING group to the same levels as controls. **(E1)** One-Way ANOVA *p* < 0.01. **(E2)** One-Way ANOVA *p* = 0.004. **(E3)** One-Way ANOVA *p* = 0.01. Multiple comparisons between groups were performed through *post hoc* Holm-Sidak. Asterisks indicates significance between groups: *p < 0.05, **p < 0.01, ***p < 0.001. **(A2–C,E1–E3)** Data are expressed as mean ± SEM.

We then asked whether the aberrant CST projections associated with poor behavioral outcome induced by P14 lesion could be recovered by early training. ET1-P14 rats were trained from P21 to learn a skilled reaching task by MST twice/day till the plateau level of learning was achieved (Figure 8A1). To investigate whether this early learning protocol could induce activity-dependent rearrangements of CST sprouting correcting the maladaptive contralesional rewiring observed after P14 lesion, we performed the same anatomical analysis described above.

We found that neither lesion size (Figure 8A2, *t*-test, *p* = 0.914) or bulk fluorescence density of dCST and vCST funiculi (Supplementary Figure S4A) were significantly affected by training (Supplementary Figures S4B,C, One-Way ANOVA, *p* = 0.101 for dCST and *p* = 0.151 for vCST). In contrast, early training significantly reduced the aberrant spontaneous sprouting present in ET1-P14 rats from either the dCST (Figure 8B, factor group × distance *p* < 0.001, ET1-P14 TRAINING vs. ET1-P14 *p* < 0.001, SAL-P14 vs. ET1-P14 TRAINING *p* = 0.643) and the vCST (Figure 8C, *p* < 0.001, ET1-P14 TRAINING vs. ET1-P14 *p* < 0.001, SAL-P14 vs. ET1-P14 TRAINING *p* = 0.702) restoring values of controls. Furthermore, a significant drop of complexity in arborization of sprouted axons (Figure 8D) in early trained ET1-P14 animals was observed across all spinal cord laminae (Figures 8E1–E3): dorsal (Figure 8E1, *p* < 0.001, ET1-P14 TRAINING vs. ET1-P14 *p* < 0.001, SAL-P14 vs. ET1-P14 TRAINING *p* = 0.839), intermediate (Figure 8E2, *p* = 0.004, ET1-P14 TRAINING vs. ET1-P14 *p* = 0.012, SAL-P14 vs. ET1-P14 TRAINING *p* = 0.610) and ventral (Figure 8E3, *p* = 0.01, ET1-P14 TRAINING vs. ET1-P14 *p* = 0.006, SAL-P14 vs. ET1-P14 TRAINING *p* = 0.610). Thus, early training is able to fully revert the mistargeted CST sprouting induced by P14 stroke.

### Early Motor Skill Training Ameliorates Long-Term Motor Impairments Upon Injury in ET1-P14

**Figure 9:**
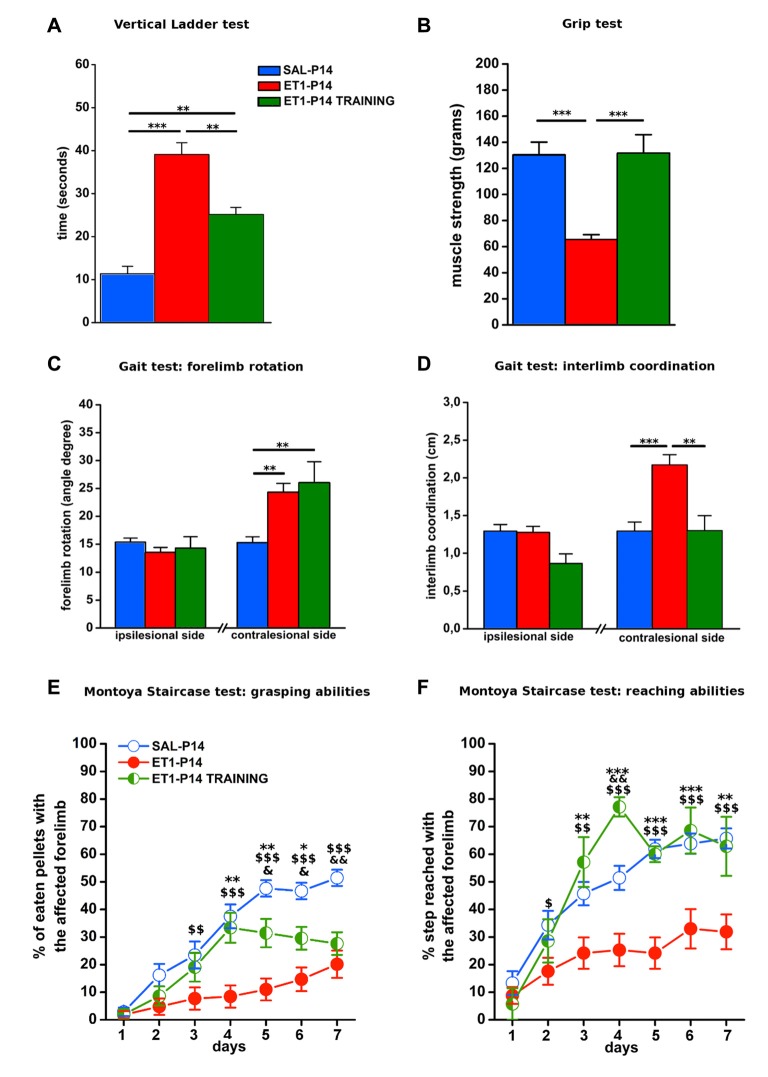
Early activity-dependent modulation of CST plasticity after ET1-P14 injury mitigates the onset of long-term motor impairments. **(A)** Partial amelioration of sensorimotor coordination promoted by early skilled motor training. **(B)** Complete recovery on both muscle strength and **(D)** interlimb coordination of lesioned side after early skilled motor training. Lack of the effect of early skilled motor training on abduction of lesioned forelimb **(C)**. Early skilled motor training partially ameliorated grasping **(E)** and fully restored reaching abilities **(F)** up to adult age. **(A–D)** One-Way ANOVA, *post hoc* Holm-Sidak. Asterisks denote significant differences between groups, **p < 0.01; ***p < 0.001. **(E,F)** Two-Way RM ANOVA, *post hoc* Holm-Sidak. Asterisks denote significant differences between ET1-P14 and ET1-P14 TRAINING, *p < 0.05; **p < 0.01; ***p < 0.001. ^$^ Denotes Significant Differences Between ET1-P14 and SAL-P14, ^$^*p* < 0.05; ^$$^*p* < 0.01; ^$$$^*p* < 0.001. ^&^Denotes significant differences between SAL-P14 and ET1-P14 TRAINING, ^&^*p* < 0.05, ^&&^*p* < 0.01. **(A–F)** Data are expressed as mean ± SEM.

To verify whether training induced rescue of axonal sprouting was associated with a better motor outcome of P14 stroke, we performed a behavioral assessment in early trained P14 stroke rats starting from P59. The same battery of tests previously described was used (Figure 8A1). We compared ET1-P14 rats with or without early training, with non-trained control groups. We found that early skilled motor training promoted a partial amelioration of sensorimotor coordination at vertical ladder climbing (Figure 9A, *p* < 0.001, ET1-P14 vs. ET1-P14 TRAINING, *p* = 0.003; ET1-P14 vs. SAL-P14, *p* < 0.001; ET1-P14 TRAINING vs. SAL-P14, *p* = 0.002), a complete recovery of both grip strength (Figure 9B, *p* < 0.001, ET1-P14 vs. ET1-P14 TRAINING, *p* < 0.001; ET1-P14 vs. SAL-P14, *p* < 0.001; ET1-P14 TRAINING vs. SAL-P14, *p* = 0.2) and of interlimb coordination (Figure 9D, *p* < 0.001, ET1-P14 vs. ET1-P14 TRAINING, *p* = 0.002; ET1-P14 vs. SAL-P14, *p* < 0.001; ET1-P14 TRAINING vs. SAL-P14, *p* = 0.98). No significant effect of motor training was detected in abduction of affected forelimb induced by lesion (Figure 9C, *p* < 0.001, ET1-P14 vs. ET1-P14 TRAINING, *p* = 0.55; ET1-P14 vs. SAL-P14, *p* = 0.002; ET1-P14 TRAINING vs. SAL-P14, *p* = 0.002). Interestingly, early motor training partially ameliorated grasping (Figure 9E, *p* < 0.001) and reaching (Figure 9F, *p* < 0.001) fine motor abilities. Indeed, ET1-P14 trained animals, similarly to controls, learned both grasping and reaching task significantly better than untrained ET1-P14 group (Figures 9E,F, factor treatment × days *p* < 0.001).

## Discussion

Although lesion timing and characteristics is considered to be a key factor for clinical outcome of developmental stroke (Eyre, 2007; Kirton, 2013; Kirton and deVeber, 2015; Dinomais et al., 2016), no study has investigated the effect of stroke age on motor function and on underlying structural plasticity mechanisms in animal models. In this study, we found that a focal cortical stroke occurring at a developmental age (P14) with high sprouting potential of CST has a dramatically worse long-term behavioral outcome than a stroke performed 1 week later when sprouting potential declines and CST pruning emerges (Hsu et al., 2006; Canty and Murphy, 2008). Indeed, early focal ischemia induces a contralesional reorganization of CST that is not present when lesion occurs at P21. Fine morphological analysis of CST sprouting pattern into the denervated spinal cord revealed that P14 stroke caused a pronounced sprouting that was targeted to inappropriate spinal cord districts that are not normally innervated by the CST. In agreement with the idea that maladaptive CST sprouting is at the basis of the worse outcome of P14 lesion, we also found that early skilled training ameliorated the fine motor impairment and the general motor activity of P14 lesioned rats and, in parallel, caused retraction of the abnormal sprouting.

Our focal ischemic lesion in developing rats recapitulated functional long-term motor impairments and the activation of CST plasticity often seen in children with hemiplegic CP (Eyre, 2007; Cioni et al., 2011; Kirton, 2013). General motor performance, assessed at adult age, was significantly affected independently from stroke age; however, P14 injured animals showed severe impairment of forelimb-hindlimb coordination that was not affected in P21 lesioned animals. Interlimb coordination is a locomotor pattern strictly dependent on the interplay between motor cortex, cerebellum and central pattern generators (CPGs) of brainstem and spinal cord (Goulding, 2009). It is interesting that locomotor behavior in rats reaches a complete maturation at about P21 (Geisler et al., 1993; Brumley et al., 2015). In this view, the presence of long-lasting interlimb coordination deficits in P14, but not in P21, lesioned animals may be sustained by stabilization of immature locomotor circuitry in place at the age of earlier lesion occurrence. Indeed, it has been suggested that perinatal brain damage affects locomotor behavior of children affected by CP by preventing the normal maturation of locomotor activity (Forssberg, 1999; Cappellini et al., 2016). An age of lesion dependent deficit was also observed for forelimb skilled abilities, such as grasping and reaching. Indeed, ET1-P14 lesioned rats showed worse skilled motor learning and lower final levels of performance than ET1-P21 lesioned animals. Previous clinical studies suggest that early lesions induce conspicuous motor deficits (Eyre et al., 2001; Eyre, 2007; Cioni et al., 2011), corroborating the importance of lesion timing as crucial factor impinging on post stroke recovery.

Our morphological investigation of motor descending pathways from the contralesional cortex in P14 and P21 lesioned rats revealed a dramatic difference in the type of rewiring induced by stroke. Indeed, lesion at P21 resulted in no contralesional sprouting of the cortico-rubral pathway, lack of pruning and limited sprouting of the ipsilaterally projecting vCST, and a nonsignificant trend for dCST sprouting. By contrast, P14 lesioned rats showed sprouting of the contralesional cortico-rubral pathway in the deafferented red nucleus, lack of vCST pruning and a dramatically widespread vCST and dCST sprouting into the denervated spinal cord. Taken together, these data indicate a massive enlargement of contralesional descending motor cortical pathways when lesion occurs at P14. Moreover, CST sprouting targets spinal cord areas that are not normally reached by CST. These rearrangements of axonal projections typical of P14 stroke are correlated with a worse behavioral outcome, strongly suggesting that P14 lesion activates mechanisms of maladaptive plasticity. The red nucleus, an important motor center involved in fine skilled abilities, was described to undergo axonal plasticity processes after different kind of lesion, related with changes of motor functions (Z’Graggen et al., 2000; Ishida et al., 2016). Thus it is conceivable that aberrant plasticity could contribute to the worse performance of P14 lesioned rats.

Previous studies in adult rodents did not observe a spontaneous contralesional axonal sprouting after unilateral focal cortical lesions (Whishaw, 2000; Starkey et al., 2012; Johnston et al., 2013; Wahl et al., 2014). However, adaptive contralesional CST plasticity has been proposed to be the main mechanism by which both pharmacological and/or rehabilitative therapies (Liu et al., 2008; Reitmeir et al., 2011; Lee et al., 2013; Lindau et al., 2014; Wahl et al., 2014) promote functional recovery in adult rodent models. Importantly, treatments promoting incorrect branching and targeting of sprouted axons have been shown to be ineffective on functional recovery (Asante and Martin, 2013; Wahl et al., 2014), demonstrating that not only the presence of axonal sprouting, but also the pattern of axonal plasticity is crucial for behavioral amelioration after stroke.

It could be speculated that the age-dependent effect of ischemic lesion on axonal sprouting could result into the triggering of a molecular program which is different between P14 and P21. Indeed, significant neural reorganization rapidly occurs at multiple brain levels at this age. For example, the molecular composition of neurotransmitter receptors in the respiratory motor nuclei changes dramatically in this narrow window of development (Kubin and Volgin, 2008). The selection of the molecular program may rely on the presence of a different transcriptional status between P14 and P21 (Li et al., 2010; Lodato et al., 2014), and could involve transcription factor expression regulation (Wang et al., 2015), expression of environmental molecular cues (Canty et al., 2006; Runker et al., 2008), and factors regulating CST myelination during development (Gibson et al., 2014). Altogether these factors could contribute to determine the pattern of sprouting in animals lesioned at different ages (Canty and Murphy, 2008).

The presence of mistargeted CST axonal sprouts in spinal cord regions not normally reached by CST could result in a negative motor outcome because of the formation of inappropriate connections between sprouted fibers and neurons located in the dorsal or ventral laminae such as propriospinal neurons (Tan et al., 2012; Azim et al., 2014), sensory excitatory interneurons (Bourane et al., 2015) and alfa-motor neurons (Yang and Lemon, 2003; Alstermark et al., 2004) normally receiving direct connection only during CST development (Maeda et al., 2016). The presence of off-target excessive spontaneous CST sprouting could be at the bases of the poor functional outcome of early lesions observed also in humans. Indeed, clinical evidence suggested that enhanced plasticity during development disturbs the normal functional refinement of CST after early brain injury (Eyre et al., 2001; Eyre, 2007; Graziadio et al., 2012). This correlation between anatomic substrate and functional outcome is corroborated by diffusion tensor imaging of gross white ipsilateral tract showing its aberrant retention after either perinatal and pediatric lesions (Kirton and deVeber, 2015; Kuo et al., 2016).

Previous studies suggested the existence of a postnatal critical time window of motor system development during which activity plays an important role in shaping CST maturation (Chakrabarty and Martin, 2005; Friel and Martin, 2007; Chakrabarty et al., 2009). In this context, modulation of CST activity-dependent plasticity by means of an early skilled motor training might induce selection of appropriate connections counteracting the maladaptive CST plasticity present at spinal cord level in P14 stroke rats. Indeed, our training protocol that boosts use-dependent development of CST during CST critical period (Joosten and Bär, 1999; Clowry et al., 2014), was sufficient to correct the maladaptive sprouting induced by P14 lesion and, importantly, promoted enduring ameliorations of both general and skilled motor abilities. In line with our results, experimental and clinical evidences suggest that motor experiences after lesion is the gold standard intervention in driving brain plasticity and therefore functional amelioration, both in adulthood and in juvenile conditions (Nudo et al., 1996; Jones et al., 2013; Wahl and Schwab, 2014; Sakzewski et al., 2015).

In summary, our work demonstrates for the first time the existence of an age-dependent regulation of CST plasticity making a 7-day difference in the age of stroke occurrence capable of resulting in remarkably different long-term motor consequences. These observations pave the way for studies revealing the intrinsic and extrinsic molecular mechanisms controlling lesion induced plasticity to implement more effective therapeutic strategies aimed at regaining or preserving motor functions.

## Author Contributions

MG and AM: conception or design of the work, data collection and analysis, data interpretation, drafting of the manuscript. NB, TP, AG and GC: conception or design of the work and drafting of the manuscript. RM: elaboration of software for data analysis and manuscript revision. CA and LG: behavioral experiments and manuscript revision.

## Conflict of Interest Statement

The authors declare that the research was conducted in the absence of any commercial or financial relationships that could be construed as a potential conflict of interest.
