# Supplementary material for: Focal Stroke in the Developing Rat Motor Cortex Induces Age- and Experience-Dependent Maladaptive Plasticity of Corticospinal System
Source: Front Neural Circuits. 2017 Jun 29;11:47. doi: 10.3389/fncir.2017.00047 (PMC5489564; doi:10.3389/fncir.2017.00047)
Supplement: Supplementary file 1 [file Image_1.pdf]

Supplementary Material

Focal stroke in the developing rat motor cortex induces age- and experience-dependent maladaptive plasticity of corticospinal system

Mariangela Gennaro*[1-2], Alessandro Mattiello*[1-2], Raffaele Mazziotti[1-2], Camilla Antonelli[3-4], Lisa Gherardini[2,5], Andrea Guzzetta[3-4], Nicoletta Berardi[1-2], Giovanni Cioni[3-4], Tommaso Pizzorusso[1-2]

* These authors contributed equally to the work

1 Department of Neuroscience, Psychology, Drug Research and Child Health NEUROFARBA, University of Florence, Area San Salvi – Pad. 26, I-50135 Florence, Italy

2 Institute of Neuroscience, National Research Council (CNR), Via Moruzzi 1, I-56124 Pisa, Italy

3 Department of Clinical and Experimental Medicine, University of Pisa, via Savi 10, I-56126 Pisa, Italy

4 Department of Developmental Neuroscience, IRCCS Stella Maris Scientific Institute, Viale del Tirreno 331, I-56128 Calambrone (Pisa), Italy

5 Institute of Clinical Physiology, National Research Council (CNR), Strada del Petriccio e Belriguardo 35, 53100 Siena Italy

Running title: Critical period for maladaptive plasticity after developmental stroke

Corresponding author address:

Institute of Neuroscience, National Research Council (CNR)

via Moruzzi 1, Pisa I-56124, Italy.

Email: tommaso@in.cnr.it

Tel: +390503153167 Fax: +390503153220

Supplementary figures

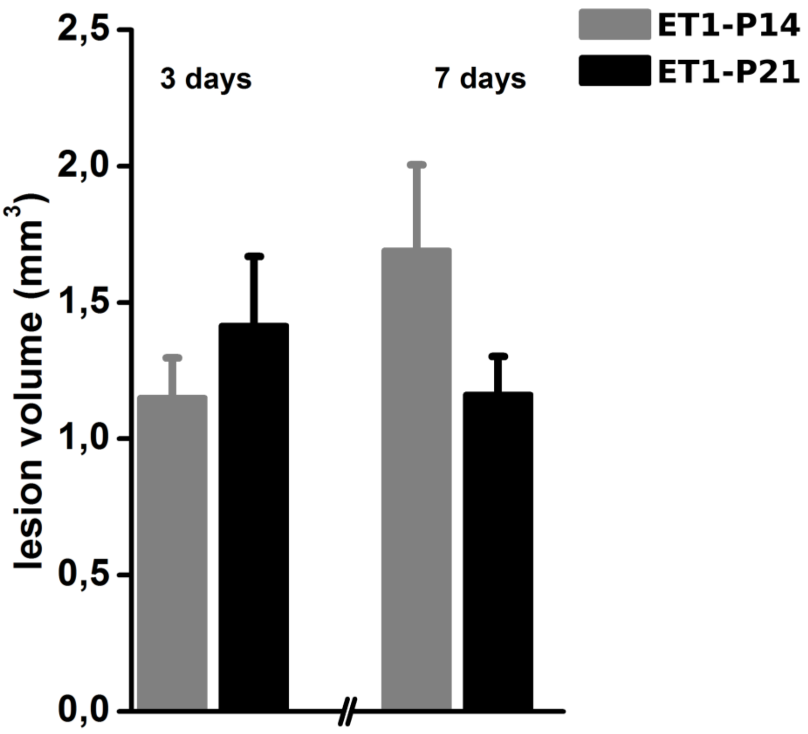

Figure S1. Ischemic lesion volume measured 3 and 7 days after injury. No statistical difference was detected between ET1-P14 and ET1-P21 groups. Two-Way ANOVA, factor age of treatment p = 0.40, factor days x age of treatment p=0.17. ET1-P14 3 days, n=3; ET1-P21 3 days, n=5; ET1-P14 7 days, n=3; ET1-P21 7 days, n=5. Data are expressed as mean ± SEM.

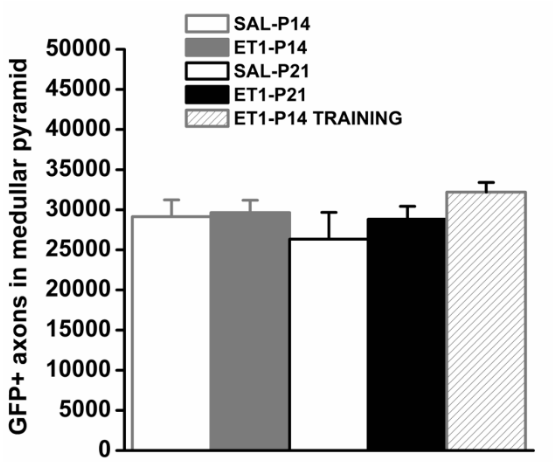

Figure S2. Number of GFP^{+} axons in the pyramidal medulla. No statistical differences between the different groups were detected, confirming that different groups showed a similar tracer

transduction efficiency. Kruskal-Wallis, p=0.435. SAL-P14 n=5, ET1-P14 n=6, SAL-P21 n=4, ET1-P21 n=6, ET1-P14 TRAINING n=5.

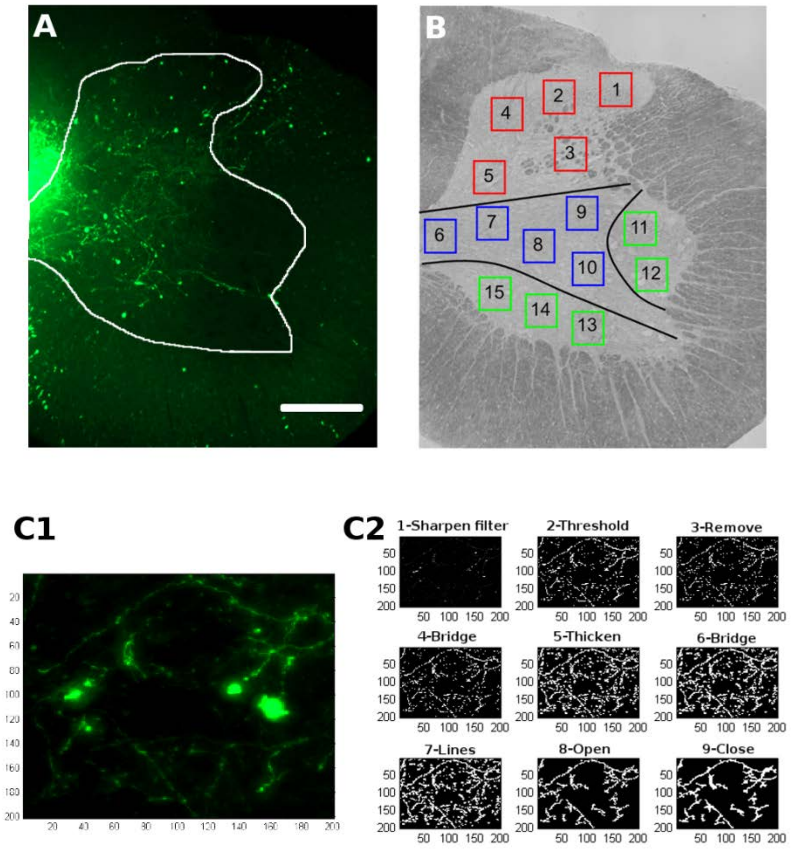

Figure S3. ROIs selection and position for complexity analysis of sprouted axons. Coronal section of a denervated C8 spinal cord in fluorescence **(A)** and brightfield **(B)** acquisition modes from a SAL-P14 animal. In **(B)** is also indicated the standardized position of ROIs for MATLAB® analysis: red squares sample dorsal laminae (1 to 6), blue squares samples intermediate lamina (7) and green squares sample ventral laminae (8-9). Scale bar: 250 µm. **(C1)** Morphological imaging processing and operations carried out on a typical sampling ROI **(C2)** using MATLAB® function “bwmorph” for the detection of axonal morphology. First, sharpen filter and thresholding were applied in order to maximize signal-to-noise ratio, then “remove” operator sets interior pixels as 0, thus leaving only pixels on the boundary. “Bridge” and “thicken” operators allow the connection of neighbouring unconnected pixels and add pixels to the exterior of objects, respectively. The

function "strel" creates structural elements of a specific shape, in this case lines on different orientation (0°, 30°, 60°, 90°). Finally, typical morphological operators such as opening (dilation followed by an erosion) and closing (erosion followed by a dilation) refine detected objects for an optimal skeletonization and extrapolation of desired parameters. For further information, see MATLAB® documentation (https://it.mathworks.com/help/matlab/) for "bwmorph" and "strel" functions.

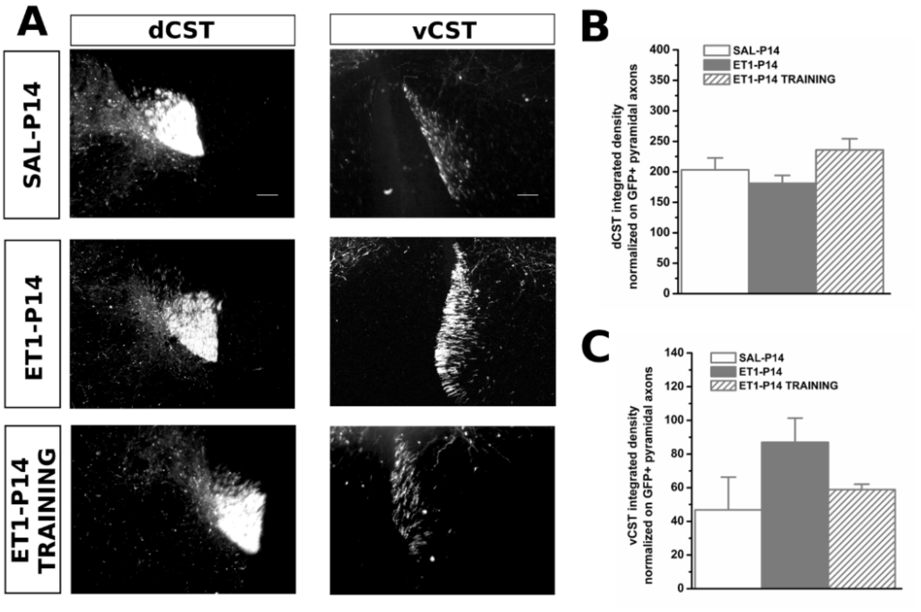

Figure S4. Early training does not promote developmental pruning of ipsilateral ventral funiculus. (A) Representative images of dCST (left) and vCST (right) from animals of different groups. Scale bar: 100 µm. **(B)** Fluorescence integrated density of dCST. **(C)** Fluorescence integrated density of vCST. No statistical difference was present for both dCST and vCST, despite an evident trend in vCST, comparing ET1-P14 TRAINING group with ET1-P14 lesioned only and relative control (One-Way ANOVA, p=0.101 for dCST and p=0.151 for vCST).

Supplementary Methods

Animals

Animals were housed with a 12 h/12 h light/dark cycle with food and water ad libitum in a standard environment (3 rats in 30 cm × 40 cm × 20 cm laboratory cages). During the assessment of skilled reaching performance, rats were fed with 20 g of Purina rat chow once a day after the daily tests (Whishaw and Pellis, 1990) in addition to the food pellets they obtained while performing the test (Bio-Serv dustless precision pellets product F0021). Rats weight was kept at about 90–100% of their expected body weight.

Profiling of motor impairment

Gait analysis was performed to assess animal locomotor pattern and forelimb posture during walking according to (de Medinaceli et al., 1982; Metz and Schwab, 2004; Parkkinen et al., 2013). Limb rotation and inter limb coordination were evaluated as described previously (Metz and Schwab, 2004). Three trials for each animal were recorded and a series of at least 10 sequential steps recorded in the same session was used to determine the mean values of each measurement.

Vertical ladder: animals were trained to climb a vertical ladder of 100 cm length with a regular arrangement (1 cm distance between rungs) (Metz and Whishaw, 2009). Three trials for each animal were video recorded and analyzed offline. Average time taken to climb the full vertical ladder length was quantified.

Grip strength test: animals were placed over a base plate of black sand-blasted Perspex, in front of a steel grasping trapeze-shape bar. Animals were then gently pulled back from the base of the tail and the maximum force exerted before they let go was measured by a dynamometer sensing the peak amount (Ugo Basile Grip-Strength meter). Forelimbs grip strength was measured as average tension force of three trials for each animal.

Skilled Reaching test: animals were trained for 7 days from P60 in Montoya staircase test (MST) apparatus (Montoya et al., 1991) to assess enduring skilled reaching impairments. Two parameters were measured: 1) number of grasped pellets; 2) step level reached on each side of the staircase during the task. Animals skilled reaching abilities were assessed once/day for 15 minutes.

Motor training

To test whether the increase of activity-dependent CST plasticity could interfere with the maladaptive CST rewiring previously observed in P14 lesioned animals, a group of ET1-P14 rats was trained twice/day until they learned the task in the MST apparatus starting one week after lesion induction (P21). Afterwards the effect of this early CST plasticity modulation on general movement performance and on reaching capability was assessed during adulthood, from P59, and the results were compared with those of control groups consisting of untrained P14 ET1/Saline injected animals.

GFP^+ axons density in the medullar pyramids

GFP^+ axons density in the medullar pyramids was performed on images acquired at a fluorescence microscope (Axio Imager.Z2, Zeiss) equipped with Apotome.2 (Zeiss). Two fields from the labelled pyramidal medulla per slice were sampled and acquired using 63x oil objective (EC-PLAN-NEOFLUAR oil objective (N.A.1.25). GFP^+ axon density was analysed offline using the software Imaris (Bitmap®): axons were detected as "spots", with a minimum diameter settled on 1 µm and a double cut-off “quality” filter (the background pixel intensity and the saturated pixels) optimized to include only reliable axons. The number of GFP^+ axons was then extrapolated to the total area of pyramid per slice, measured from 5x magnification (EC-PLAN-NEOFLUAR objective, N.A. 0.25) images acquired from the same set-up.

Analysis of laminar pattern distribution of sprouted axons

The analysis of laminar pattern distribution of GFP^+ sprouted axons was performed using a custom-made MATLAB® algorithm, based on *mathematical morphology* theory. 15 square ROIs (129x129 µm) per section were selected on standardized positions across all laminae of dorso-ventral spinal cord using brightfield cues. Images processing consisted in background subtraction, application of sharpen filter and binarization on each ROI. Sharpen radius (size of the region around the edge pixels that is affected by sharpening) was maintained constant (15), whereas sharpen amount (strength of the sharpening effect) ranges from 0.1 to 3, depending on imaging quality. Afterwards, MATLAB *"bwmorph"* function carried out several mathematical operations on morphology of detected objects ("remove", "bridge", "thicken", "close"). To perform branchpoints and endpoints computation, the image was further skeletonized and shrunk, using the same MATLAB *"bwmorph"* function. In this way, it was possible to calculate the number of objects per ROI and to extrapolate several parameters: number of branchpoints and endpoints, complexity (sum of branchpoints and endpoints per object), area (total number of pixel per object), perimeter, length (the number of pixel of the major axis of an ellipse containing the object), orientation (angle between X axis and the major axis of an ellipse containing the object), fluorescence intensity. For laminar pattern axons distribution, we used "*axonal complexity index*" which was computed as:

$$\textit{Axonal Complexity Index} = Na * Ca$$

where *Na* is the mean number of detected axons per laminae (normalized for the number of GFP^+ axons in medullar pyramids) and *Ca* is the mean axonal complexity per laminae.

Supplementary Methods References

de Medinaceli, L., Freed, W.J., and Wyatt, R.J. (1982). An index of the functional condition of rat sciatic nerve based on measurements made from walking tracks. Exp Neurol *77*, 634-643.

Metz, G.A., and Schwab, M.E. (2004). Behavioral characterization in a comprehensive mouse test battery reveals motor and sensory impairments in growth-associated protein-43 null mutant mice. Neuroscience *129*, 563-574.

Metz, G.A., and Whishaw, I.Q. (2009). The ladder rung walking task: a scoring system and its practical application. J Vis Exp.

Montoya, C.P., Campbell-Hope, L.J., Pemberton, K.D., and Dunnett, S.B. (1991). The "staircase test": a measure of independent forelimb reaching and grasping abilities in rats. J Neurosci Methods *36*, 219-228.

Parkkinen, S., Ortega, F.J., Kuptsova, K., Huttunen, J., Tarkka, I., and Jolkkonen, J. (2013). Gait impairment in a rat model of focal cerebral ischemia. Stroke Res Treat *2013*, 410972.

Whishaw, I.Q., and Pellis, S.M. (1990). The structure of skilled forelimb reaching in the rat: a proximally driven movement with a single distal rotatory component. Behav Brain Res *41*, 49-59.
